# Tyrosinase@HKUST-1: a super stable biocatalyst efficient for catecholic product synthesis

**DOI:** 10.1186/s40643-021-00462-7

**Published:** 2021-10-27

**Authors:** Xiao-Feng Lü, Chao-Yun Feng, Shuangfei Li, Guo-Hao Liu, Zhen Yang

**Affiliations:** 1 College of Life Sciences and Oceanography, Shenzhen Key Laboratory of Microbial Genetic Engineering, Shenzhen University, 1066 Xue Yuan Avenue, Shenzhen, 518055 Guangdong China; 2 College of Life Sciences and Oceanography, Shenzhen Key Laboratory of Marine Bioresources and Eco-Environmental Science, Shenzhen University, Shenzhen, 518055 Guangdong China

**Keywords:** Tyrosinase, HKUST-1, Metal–organic frameworks (MOFs), Catecholic products, *o*-Diphenols, Enzyme immobilization, Hydroxytyrosol, l-DOPA

## Abstract

Although metal–organic frameworks (MOFs) have been considered as promising matrices for enzyme immobilization, HKUST-1, constructed from copper acetate (CuAc_2_) and benzene 1,3,5-tricarboxylate (BTC), has rarely been explored for this application. In this study, mushroom tyrosinase (EC 1.14.18.1) was immobilized in the form of tyrosinase@HKUST-1 following a simple reaction procedure by mixing BTC with the enzyme prior to addition of CuAc_2_. The resultant biocatalyst was characterized in both structural features and catalytic properties. Upon incorporation into the HKUST-1 frameworks, the enzyme gained a prominent enhancement in stability against pH, temperature and storage: When incubated at 50 °C and pH 6.0, tyrosinase@HKUST-1 presented a half-life of 32.6 h, which is 77-fold and over tenfold higher than that of the free enzyme and its other immobilization forms, respectively; and the catalyst fully maintained its activity for at least 2 months when stored at 30 °C. The applicability of this new biocatalyst was demonstrated by employing it as catalyst for regioselective *ortho*-hydroxylation reactions to produce catecholic products with huge pharmacological effects, i.e., hydroxytyrosol and l-DOPA, with excellent yields and productivities. This study has thus offered a facile immobilization method to prepare a novel biocatalyst with super stability, and tyrosinase@HKUST-1 so formed from crude mushroom extract provides an efficient catalyst which can be applied to the production of catecholic products with health benefits.

**Graphical Abstract:**

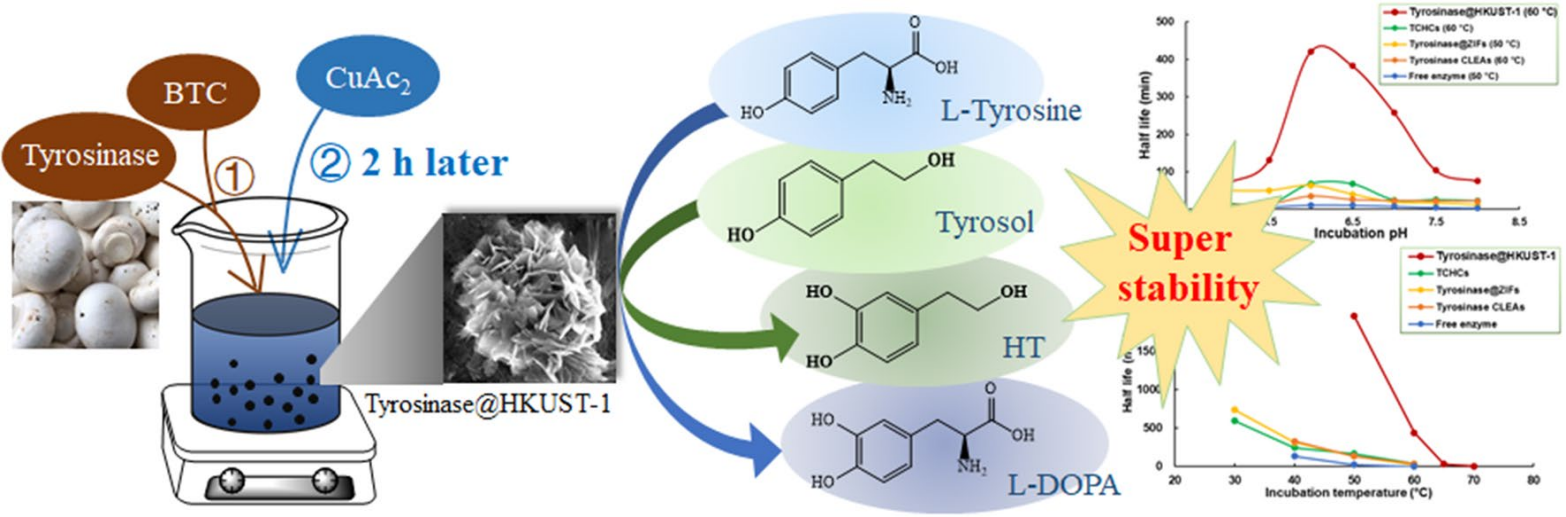

## Introduction

As an emerging class of porous materials with a broad spectrum of applications which are ever-expanding, MOFs have gained increasing attention and interests (Yuan et al. 2018). In particular, MOFs have been recognized as novel matrices for efficient enzyme immobilization due to their fascinating structural and functional tunability, easy immobilization processing, and excellent catalytic performance presented by the enzyme–MOF hybrid composites (for reviews see (Vaidya et al. 2020; Liang et al. 2020)). Among them is HKUST-1, a highly porous metal coordination polymer with a molecular formula of [Cu_3_(BTC)_2_(H_2_O)_n_] (BTC = benzene-1,3,5-tricarboxylate), which was first reported by Chui et al. (1999).

Although being one of the most extensively studied MOFs for applications such as gas separation and storage (Al-Janabi et al. 2015), HKUST-1 has rarely been explored for its potentials in enzyme immobilization. So far there are only two reports on this aspect. Nobakht et al. (2018) have tried to immobilize porcine pancreatic lipase (PPL) using HKUST-1 as the support, but the stability of the immobilized enzyme was only mildly improved as compared to that of the free enzyme. Zhang et al. (2020) have successfully prepared laccase@HKUST-1 biocomposites which are more active and stable than the free enzyme and have shown potential for degradation of bisphenol A.

This study was aimed to immobilize a new enzyme, mushroom tyrosinase (polyphenol oxidase, EC 1.14.18.1), as tyrosinase@HKUST-1 hybrid composites by following a simple reaction procedure we developed. Tyrosinase is a copper-containing oxidoreductase catalyzing the hydroxylation of monophenols to *o*-diphenols and their subsequent oxidation to *o*-quinones (Robb 1984). This enzyme has found promising potential in broad applications such as wastewater treatment (Atlow et al. 1984; Xu and Yang 2013), biosensor design (Nistor et al. 1999; Wang et al. 2015; Lu et al. 2016), synthesis of pharmaceuticals and nutraceuticals (Pialis and Saville 1998; Cheng et al. 2018; Wei et al. 2020b), and prodrug activation for cancer therapy (Lian et al. 2018). Our previous work has demonstrated that this enzyme, when immobilized as cross-linked enzyme aggregates (CLEAs) (Xu et al. 2011), tyrosinase–Cu_3_(PO_4_)_2_ hybrid composites (TCHCs) (Wei et al. 2020b), and tyrosinase@ZIF-8 (here ZIF = zeolitic imidazolate framework, a type of MOFs) (Wei et al. 2021), had its stability significantly enhanced as compared to the free enzyme.

The tyrosinase@HKUST-1 hybrid composites produced in this current study were found to present a super stability against pH, temperature, storage and recycling. This new biocatalyst was also applied to the synthesis of catecholic products as a first attempt to explore its application potential.

## Materials and methods

### Materials

l-Tyrosine was purchased from Sigma-Aldrich China Inc. Ammonium persulfate (APS), N,N,N’,N’-tetramethylethylenediamine (TEMED) were purchased from Sangon Biotech (Shanghai) Co., Ltd. Acrylamide/bisacrylamide solution (29:1) was from Labgic Technology Co., Ltd. l-3,4-dihydroxyphenylalanine (l-DOPA), benzene 1,3,5-tricarboxylate (BTC), copper acetate (CuAc_2_) monohydrate, tyrosol, hydroxytyrosol (HT), l-ascorbic acid, and all other chemicals were obtained from Shanghai Macklin Biochemical Co., Ltd. Crude tyrosinase solution was prepared from fresh mushrooms (*Agaricus bisporus*) as described in (Yang and Robb 1993).

### Preparation and characterization of tyrosinase@HKUST-1

BTC (9.70 g) was added into 1 L crude enzyme solution, homogenized for 1 min, followed by addition of 77 mL NaAc–HAc buffer (200 mM, pH 7.0). The suspension was then mixed with a magnetic stirrer at 12 °C for 2 h before 77 mL of 90 mM CuAc_2_ solution in the acetate buffer was added. The mixture was then stirred at 12 °C for 8 h. The resultant product was obtained after centrifugation at 10,000 rpm for 10 min, washing at least three times with acetate buffer, and vacuum drying at room temperature overnight. The produced tyrosinase@HKUST-1 was characterized, in comparison to the enzyme-free HKUST-1, by using X-ray diffraction (XRD, D8 ADVANCE, Bruker AXS, Germany), Fourier transform infrared spectroscopy (FT-IR, Nicolet 6700, Thermo Fisher Scientific, USA), scanning electron microscopy (SEM) and energy-dispersive X-ray diffraction spectroscopy (EDS) (APREO, Thermo Fisher, USA).

### Preparation of tyrosinase@HKUST-1@PAAm and tyrosinase@PAAm

Tyrosinase@HKUST-1 was further entrapped into polyacrylamide (PAAm) gel by adding 120 mg pulverized powders of the catalyst, 7.5 mL phosphate buffer (pH 6.0, 50 mM), 2.5 mL 30% acrylamide/bisacrylamide solution (29:1), 100 $\upmu$L 10% APS solution and 10 $\upmu$L TEMED, in order, to a 15 mL test tube with a dimension of 1.5 cm (diameter) × 9 cm (length). After thorough mixing, the suspension was allowed to stay for polymerization at room temperature for 1 h. The resultant cylindrical gel (tyrosinase@HKUST-1@PAAm) was then released from the test tube. After cleaning, it was carefully cut into 12 pieces of equal size, each with a weight of ~ 0.75 g, followed by being stored at 4  C in phosphate buffer.

Tyrosinase@PAAm was prepared in the same way by mixing 6.0 mL crude enzyme solution, 1.5 mL phosphate buffer, 2.5 mL 30% acrylamide/bisacrylamide solution (29:1), 100 $\upmu$L 10% APS solution and 10 $\upmu$L TEMED.

### Activity and stability assays

Activity: tyrosinase activity was assayed spectrophotometrically by following the oxidation of l-DOPA to dopachrome, which was reflected by an increase in the absorbance at 475 nm (Yang and Robb 1993). Typically, 5.0 mg of tyrosinase@HKUST-1 was added to 10 mL of sodium phosphate buffer (50 mM, pH 6.0) containing 0.8 mM l-DOPA in a beaker, which was placed in a shaking incubator at 30 °C with agitation of 200 rpm. After reaction for 10 min, a 2-mL sample was taken for absorbance reading at 475 nm as a measure of the initial reaction rate. All tests were carried out at least in triplicate with the coefficient of variation (% CV) not greater than 5% and 10% for the free and immobilized enzyme, respectively.

Stability against pH and temperature: the residual activity of the enzyme was determined after incubation for a specified period at various pH’s (pH 5–8) and temperatures (30–70 °C).

Recyclability: the immobilized enzyme was subjected to 10-min activity assay as described above. Then the catalyst was collected by centrifugation, followed by washing twice with phosphate buffer. Fresh substrate solution was then added for the next-round reaction.

Storage stability: the storage stability of the free and immobilized enzyme was examined by comparing the residual activity of the enzyme, free or immobilized, after being stored at 30 °C, in air or in phosphate buffer (50 mM, pH 6.0), for a certain period.

### Synthesis of hydroxytyrosol (HT) from tyrosol

For a typical reaction, the substrate solution containing 2 mM tyrosol and 55 mM l-ascorbic acid in 10 mL phosphate buffer (200 mM, pH 6.6) was placed in a shaking incubator with agitation of 250 rpm at 30 °C. 40 mg of tyrosinase@HKUST-1 was added to start the reaction, and every 30 min samples were taken for HPLC analysis.

### Synthesis of l-DOPA from l-tyrosine

A typical reaction was carried out by adding 50 mg of tyrosinase@HKUST-1 to the substrate solution containing 2 mM L-tyrosine and 30 mM l-ascorbic acid in 10 mL of sodium phosphate buffer (100 mM, pH 7.0), with agitation of 250 rpm at 30 °C. Every 30 min samples were taken for HPLC analysis.

### HPLC analysis

HPLC analysis for the above two synthetic reactions was performed on a Shimadzu LC-16 HPLC system equipped with an SPD-16 UV/vis detector and a 150 mm × 4.6 mm, 5 μm Inertsil ODS-SP column (GL Sciences Inc. Japan). For detection of tyrosol and hydroxytyrosol, a solvent mixture of 0.1% formic acid / 25% methanol was employed as the mobile phase with a flow rate of 1.0 ml/min, and the absorbance at 278 nm was followed within 10 min. The HPLC analysis of l-tyrosine and l-DOPA was run with the mobile phase of H_2_O/CH_3_OH/H_3_PO_4_ (979.5:19.5:1 by volume, pH 2.0), as described in Xu et al. (2012).

### TLC detection

Tyrosol and hydroxytyrosol were detected by running the thin-layer chromatography (TLC) plates with a solvent mixture of hexane/ethyl acetate/formic acid (60:40:2 by volume), followed by spraying with the vanillin reagent (2.5 g vanillin dissolved in an ethanol solution containing 10% (v/v) concentrated sulfuric acid).

## Results and discussion

### Characterization of tyrosinase@HKUST-1

**Fig. 1:**
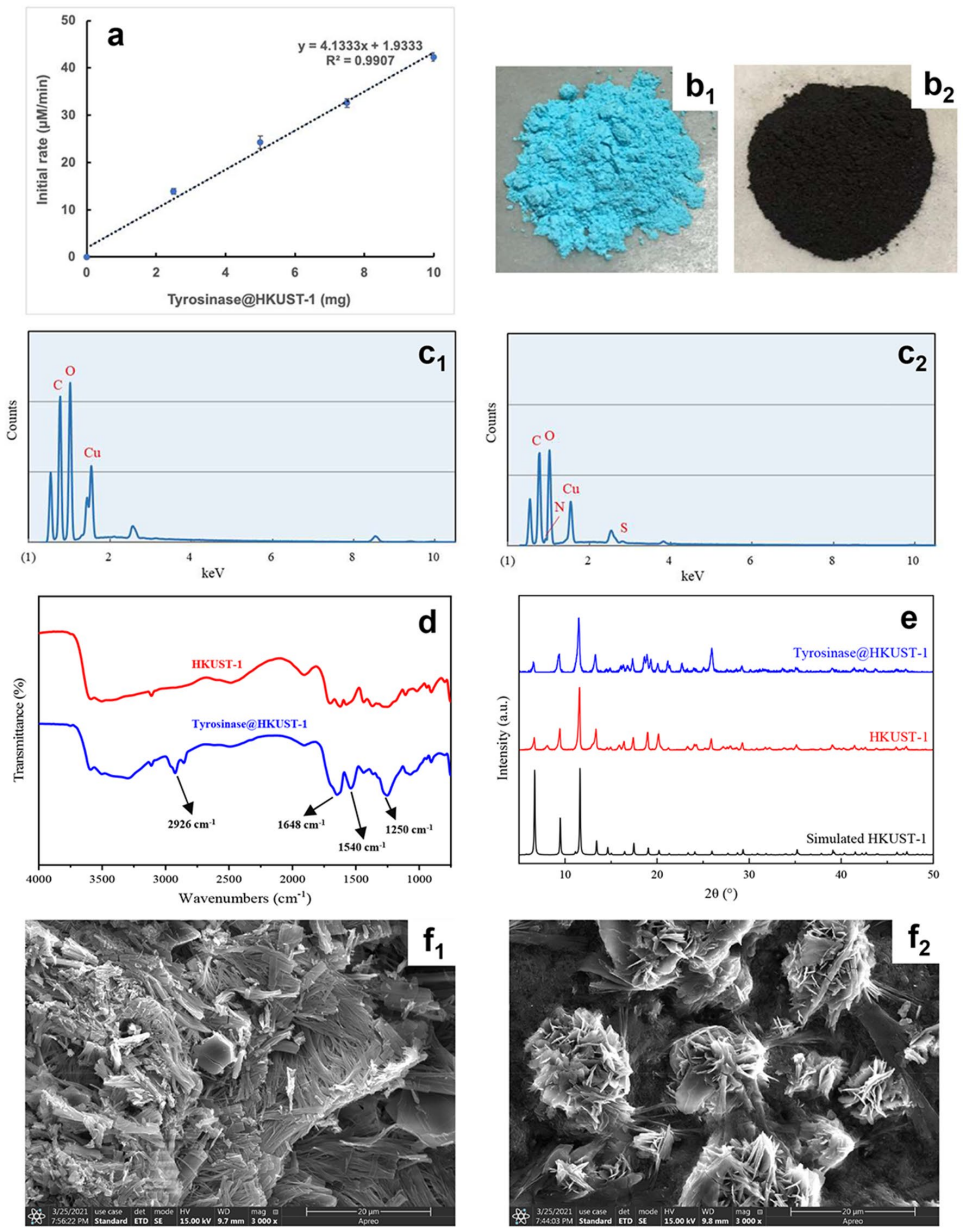
Characterization of tyrosinase@HKUST-1: (**a**) activity assay; (**b**) photos of HKUST-1 (b1) and tyrosinase@HKUST-1 (b2); (**c**) EDS images of HKUST-1 (c1) and tyrosinase@HKUST-1 (c2); (**d**) FT-IR spectra of HKUST-1 and tyrosinase@HKUST-1; (**e**) XRD images of HKUST-1 and tyrosinase@HKUST-1 as compared to that of the simulated HKUST-1; (**f**) SEM images of HKUST-1 (f1) and tyrosinase@HKUST-1 (f2)

Tyrosinase@HKUST-1 was prepared in aqueous solution under mild conditions by following the procedures described above in the Experimental Section. It has shown to be catalytically active. As displayed in Fig. 1a, its activity was correlated proportionally with the amount of the immobilized enzyme applied.

While the enzyme-free HKUST-1 powders are blue (Fig. 1, b1), the tyrosinase@HKUST-1 composites are black (Fig. 1, b2). The EDS study (Fig. 1, c1, c2) provides evidence that the enzyme molecules have been incorporated into the HKUST-1 frameworks, as in the hybrid product there are new elements (N and S), which could come solely from protein molecules. The firm incorporation of the enzyme into the HKUST-1 frameworks was also ascertained by FT-IR analysis (Fig. 1d): The FT-IR spectrum of tyrosinase@HKUST-1 shows characteristic bands of the enzyme (1648, 1540, 1250 cm^−1^, corresponding to C=O stretching vibration, N–H bending vibration and C–H stretching vibration of the protein’s amide bond, respectively). Similar IR spectra were also observed for lipase@HKUST-1 (Nobakht et al. 2018) and laccase@HKUST-1 (Zhang et al. 2020).

The XRD patterns of both HKUST-1 and tyrosinase@HKUST-1 we prepared showed no visible difference (Fig. 1e), both matching well with that of the simulated HKUST-1 with characteristic peaks at 2θ ≈ 6.5^o^, 9.5^o^, 11.5^o^ and 13.4^o^ (Huo et al. 2013). This indicates that the structural integrity and crystallinity of the HKUST-1 crystals were highly preserved when the enzyme was encapsulated.

The morphologies of HKUST-1 (Fig. 1, f1) and tyrosinase@HKUST-1 (Fig. 1, f2) visualized by SEM look quite different: The enzyme-free HKUST-1 showed the bar-shaped structure, while tyrosinase@HKUST-1 formed microflowers with size distribution ranging between 5–20 μm. Similar flower-like SEM images were also observed for laccase@HKUST-1 but with smaller sizes (1–1.5 μm) (Zhang et al. 2020). As suggested by the authors (Zhang et al. 2020), the morphology of the enzyme@HKUST-1 composites different from that of the enzyme-free HKUST-1 crystals may be due to the aggregative growth kinetics mediated by the enzyme (and the substances contained in the crude enzyme solution in our case). Nevertheless, the microflower structure with a highly exposed surface area appearing in the hybrid biocatalyst is beneficial as it facilitates the substrate accessibility to the enzyme.

### Super stability presented by tyrosinase@HKUST-1

**Fig. 2:**
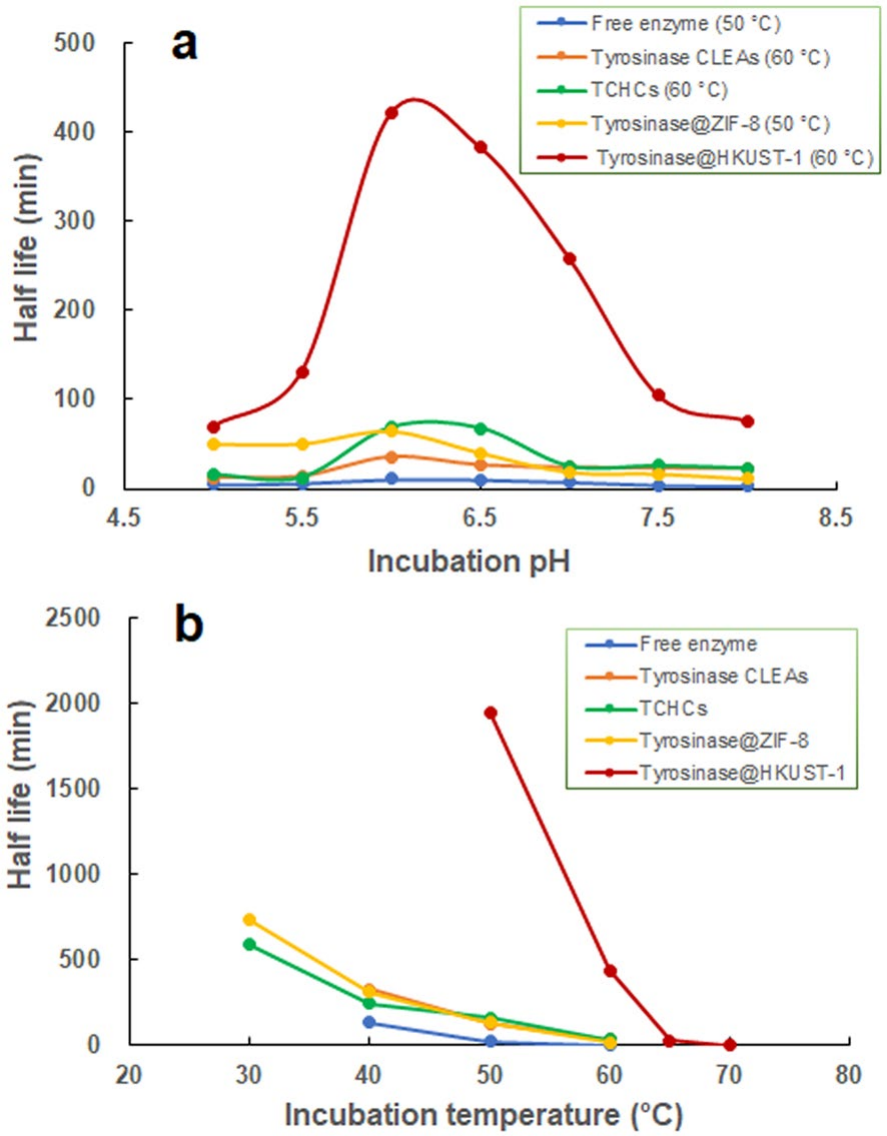
A comparison of the stability of tyrosinase@HKUST-1 against pH (**a**) and temperature (**b**) with that of the free enzyme and its other three immobilization forms

Figure 2 presents a comparison of the stability of tyrosinase@HKUST-1 against both pH and temperature to that of the free enzyme and its three other immobilization forms prepared in our laboratory: tyrosinase CLEAs (Xu et al. 2011), TCHCs (Wei et al. 2020b), and tyrosinase@ZIF-8 (Wei et al. 2021).

As shown in Fig. 2a, encapsulation into HKUST-1 did not alter the enzyme’s optimal pH for incubation (which is pH 6.0), but when incubated at 60 °C and pH 6.0, tyrosinase@HKUST-1 achieved a long half-life of 7.0 h, which was 5.1-, 5.6-, 10.9- and 37.9-fold higher than those obtained by TCHCs (60 °C), tyrosinase@ZIF-8 (50 °C), tyrosinase CLEAs (60 °C) and the free enzyme (50 °C), respectively.

The thermostability of tyrosinase@HKUST-1 was also dramatically higher than that of the free enzyme and its other immobilization forms (Fig. 2b). Take the half-lives obtained at 50 °C as an example for comparison, tyrosinase@HKUST-1 exhibited a superb half-life of 32.6 h, which was 11.8-, 14.5-, 14.7-, and 78.1-fold as high as those obtained by TCHCs, tyrosinase@ZIF-8, tyrosinase CLEAs and the free enzyme, respectively. In support of our findings, Zhang et al. (2020) have also observed a higher thermostability for laccase@HKUST-1 than that for laccase CLEAs. This may be explained by the fact that the HKUST-1 crystal matrices provide a more rigid structure and hence stronger protection to the enzyme than the cross-linked aggregates, thus preventing the enzyme molecules from conformational changes and in turn a loss in enzyme activity.

**Fig. 3:**
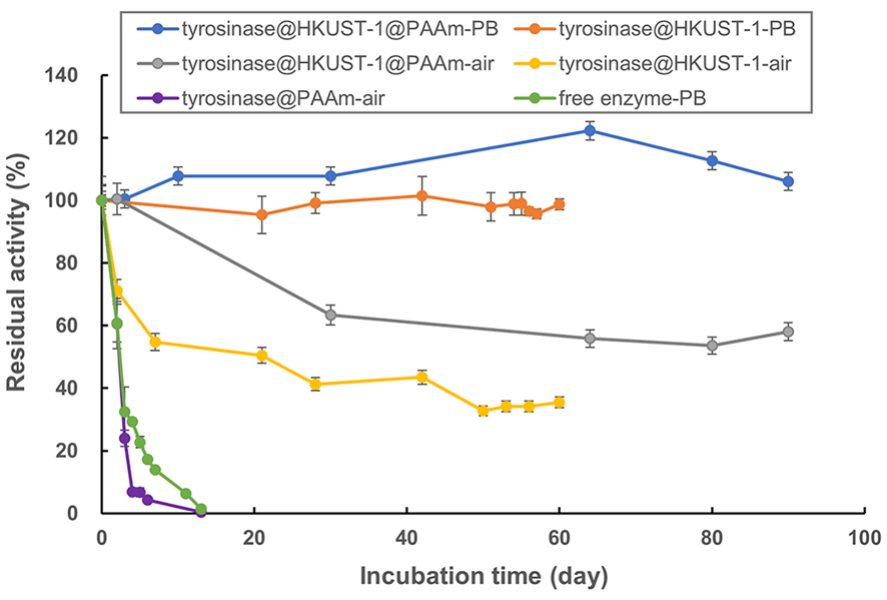
Storage stability of tyrosinase@HKUST-1 at 30 °C. In the labels, “PB” or “air” as the suffix refers to phosphate buffer (50 mM, pH 6.0) or air, respectively, in which the catalyst was incubated for storage

Tyrosinase@HKUST-1 also possessed an excellent storage stability. As shown in Fig. 3, upon incubation in phosphate buffer at 30 °C, the activity of tyrosinase@HKUST-1 remained fairly unchanged for at least 2 months (after that the tests were terminated), whereas the free enzyme completely lost its activity within 13 days. Interestingly, when incubated in air however, tyrosinase@HKUST-1 had its activity dropped to 35.5% of its original after 60 days of storage at the same temperature. A similar contrast was also observed when tyrosinase@HKUST-1 was further entrapped into polyacrylamide (PAAm) gel (i.e., tyrosinese@HKUST-1@PAAm), and the reason is uncertain yet which deserves a further investigation.

It is worth noting that the storage stability of our catalyst is much superior relative to that for lipase/HKUST-1 (Nobakht et al. 2018) and laccase/HKUST-1 (Zhang et al. 2020), the only two examples of enzyme immobilization with HKUST-1 as the support: While tyrosinase@HKUST-1 maintained full activity after storage at 30 °C for more than 60 days, the latter two retained 80% and 70% of their original activities after storage at 4 °C for only 20 days and 30 days, respectively. Tyrosinase@HKUST-1 is even more stable than the same enzyme immobilized as TCHCs (tyrosinase–Cu_3_(PO_4_)_2_ hybrid composites), as the latter had its activity dropped to 77% of its original after 30 days of storage at 30 °C (Wei et al. 2020b). This may suggest that the HKUST-1 metal–organic coordination frameworks should possess a more stable structure than the inorganic Cu_3_(PO_4_)_2_ crystals, thus providing stronger protection to the enzyme.

**Fig. 4:**
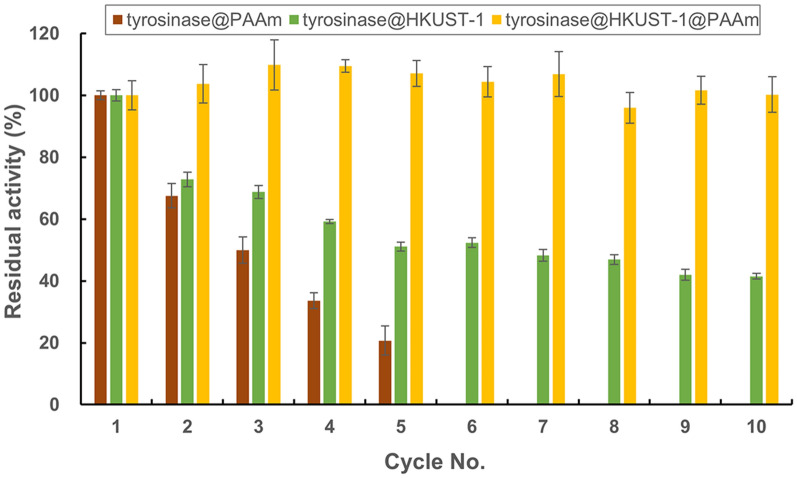
Recyclability of tyrosinase@HKUST-1

Although tyrosinase@HKUST-1 has shown to exhibit excellent catalytical performance, especially in terms of stability, it has to be admitted that this new biocatalyst was not well recyclable: almost half of its activity was lost after only 5 reaction cycles (Fig. 4), and the major reason is because the biocatalyst particles are too fine to be recovered for reuse. Our recent studies have proven that although the other three immobilization forms of mushroom tyrosinase (i.e., tyrosinase CLEAs, TCHCs and tyrosinase@ZIF-8) also did not behave well in recycling, a combination of them with other traditional immobilization methods such as entrapment into alginate gel offers a great solution for improving the catalyst’s robustness and operability (Xu et al. 2012; Wei et al. 2020b, 2021). In this study, tyrosinase@HKUST-1 was endeavored to be entrapped into polyacrylamide gel. Indeed, the resultant tyrosinase@HKUST-1@PAAm was markedly more stabilized, fully maintaining its activity after storage for at least 3 months (Fig. 3) and after reaction for at least 10 cycles (Fig. 4). It is worth noting that here the stability enhancement was more attributed to the HKUST-1 frameworks rather than to the polyacrylamide gel. This is well illustrated when comparing the storage stability (Fig. 3) and recyclability (Fig. 4) of tyrosinase@PAAm to those of both tyrosinase@HKUST-1 and tyrosinase@HKUST-1@PAAm.

### A discussion about the preparation and its role in the stability of the so-formed catalyst

In our opinion, the super stability presented by our tyrosinase@HKUST-1 relative to that of the other two enzymes both immobilized on HKUST-1, i.e., lipase@HKUST-1 (Nobakht et al. 2018) and laccase@HKUST-1 (Zhang et al. 2020), is very much attributed to the different procedures used for their preparations.

Our preparation of tyrosinase@HKUST-1 was carried out by mixing the enzyme with BTC first, homogenizing for 1 min and then magnetic stirring for 2 h, followed by addition of CuAc_2_. Several trials have been done with results showing that direct addition of CuAc_2_ to the mixture of BTC + enzyme led to the production of catalysts with rather poor stability. Therefore, we figure that mixing BTC with the enzyme for 2 h prior to addition of CuAc_2_ is critical to ensure the generation of super stable enzyme@HKUST-1 biocomposites. BTC may play the role as a cross-linker to the enzyme by having its three carboxylic groups interacting with the enzyme through electrostatic and H-bonding interactions. In particular, for tyrosinase with copper ions at its active site, the carboxylic groups of BTC might also have the possibility of interacting with the enzyme through coordination; however whether this is good or bad for the enzyme performance remains to be examined.

On the contrary, laccase@HKUST-1 was prepared by mixing the enzyme with CuAc_2_ before BTC was introduced (Zhang et al. 2020). This might be taken as a support of our assumption above, and a higher stability would be expected if the order of adding BTC and CuAc_2_ were reversed.

Lipase@HKUST-1, on the other hand, was prepared by adding the prior produced HKUST-1 crystal powders to the lipase solution (Nobakht et al. 2018). Therefore, the enzyme was immobilized on the support only through physical adsorption, and the enzyme molecules may be only attached on the outer surface of the HKUST-1 crystals. The weak interactions between the enzyme and the support may directly account for the poor stability of lipase@HKUST-1 reported in Nobakht et al. (2018).

It is worth mentioning that although BTC is a tricarboxylic acid and hence HKUST-1 may be acid-sensitive, our preparation of tyrosinase@HKUST-1 was actually done under acidic conditions (final pH of the preparation mixture was recorded to be 4.5). All the above results have shown that regardless of being subjected to this acidic treatment, the immobilized enzyme remained active and highly stable.

### Use of tyrosinase@HKUST-1 as catalyst for catecholic product synthesis

**Scheme 1:**
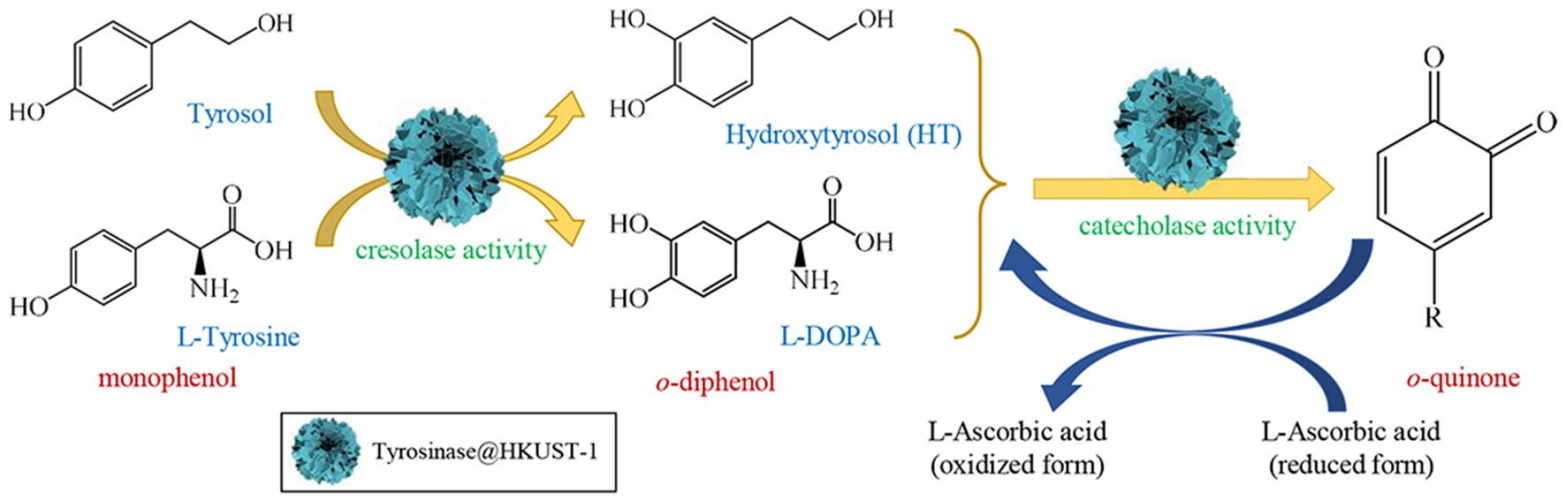
The reaction scheme for the enzymatic synthesis of catecholic products catalyzed by tyrosinase@HKUST-1

In order to demonstrate its applicability, tyrosinase@HKUST-1 was employed as catalyst for the production of two catecholic products: hydroxytyrosol (HT) and l-DOPA (Scheme 1). HT is one of the most potent natural antioxidants (Britton et al. 2019) conferring a variety of pronounced health benefits such as anti-cancer, anti-inflammatory, and anti-carcinogenic properties, just to name a few, and hence has been considered an excellent food supplement by nutraceutical and food industries (Bertelli et al. 2020). l-DOPA, on the other hand, is a drug of choice for the treatment of Parkinson’s disease (Nagatsu and Sawadab 2009). There has been a huge market requirement for these catecholic products, and production by means of biocatalysts appears as the optimal direction for cost-effective bio-based industrial processes (Achmon and Fishman 2014; Britton et al. 2019).

Our previous research has demonstrated that tyrosinase is capable of producing catecholic products such as l-DOPA (Xu et al. 2012; Wei et al. 2020b), 4-dihydroxyphenylacetic acid (Cheng et al. 2018), piceatannol (Cheng et al. 2018; Wei et al. 2020b), and 3′-hydroxypterostilbene (Cheng et al. 2018; Wei et al. 2020a) from their corresponding monophenol precursors. The rationale behind is the specific regioselectivity of the enzyme (Scheme 1): It catalyzes the conversion from a monophenol to an *o*-diphenol (cresolase activity) and subsequently to an *o*-quinone (catecholase activity); with the aid of a strong reductant such as l-ascorbate, the *o*-quinone can be reduced back to its *o*-diphenol form (catecholic product), leaving it as the sole product.

**Fig. 5:**
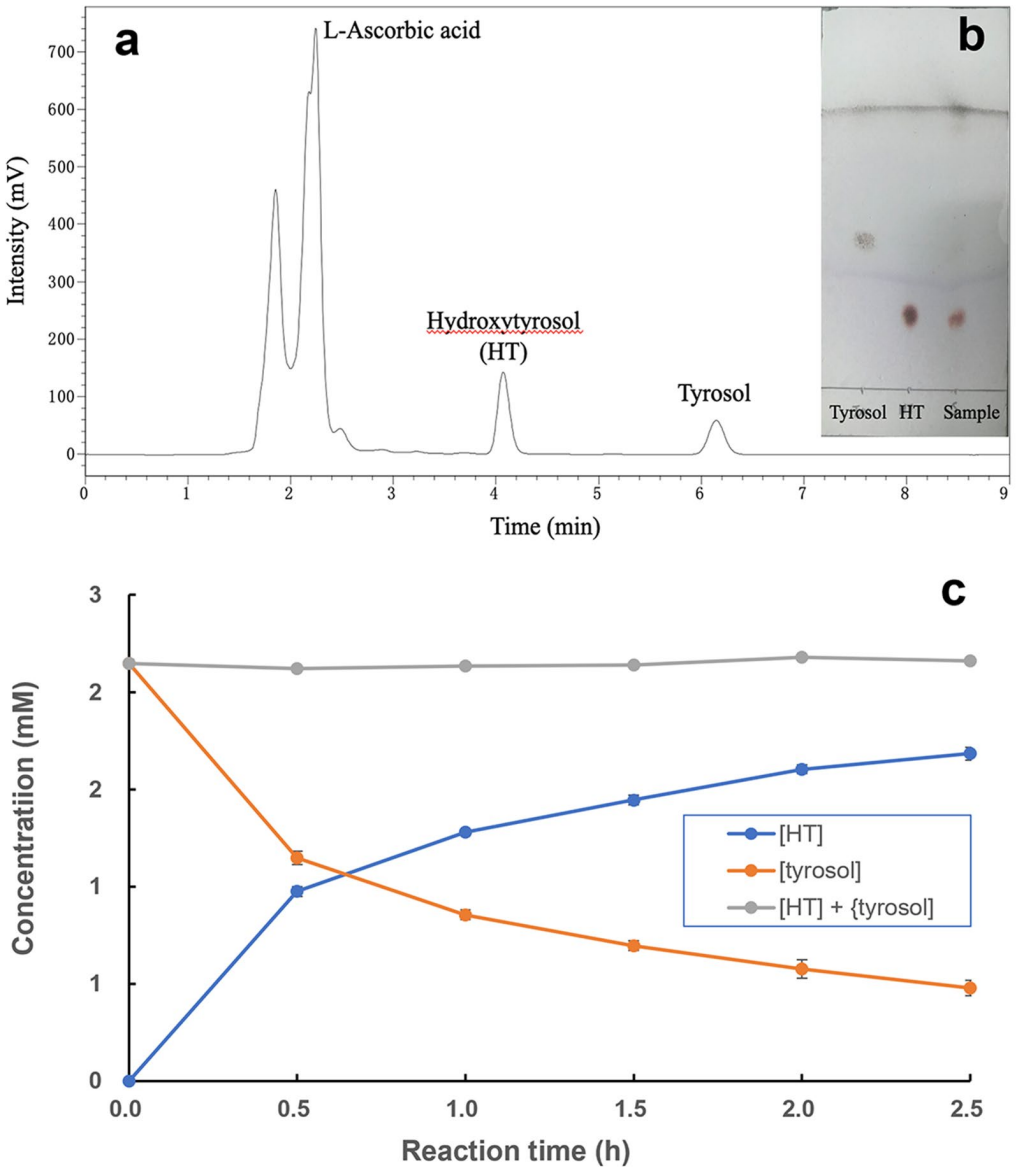
Production of hydroxytyrosol (HT) from tyrosol catalyzed by tyrosinase@HKUST-1 (I): identification of the substrate and product by means of HPLC (**a**) and TLC (**b**); time courses of the synthetic reaction (**c**)

The successful production of HT from tyrosol through *ortho*-hydroxylation, catalyzed by tyrosinase@HKUST-1, was verified by HPLC (Fig. 5a) and TLC analyses (Fig. 5b). A typical time course of the synthetic reaction is presented in Fig. 5c. The product (HT) was gradually formed, concomitant with the depletion of the substrate (tyrosol), leaving the sum of the concentrations of the two to remain fairly constant (equal to the original input of the substrate). This confirms that the new biocatalyst was effective for the synthesis and that the reaction is highly selective, with HT as the only product in the presence of a sufficient supply of the reductant, l-ascorbic acid.

**Fig. 6:**
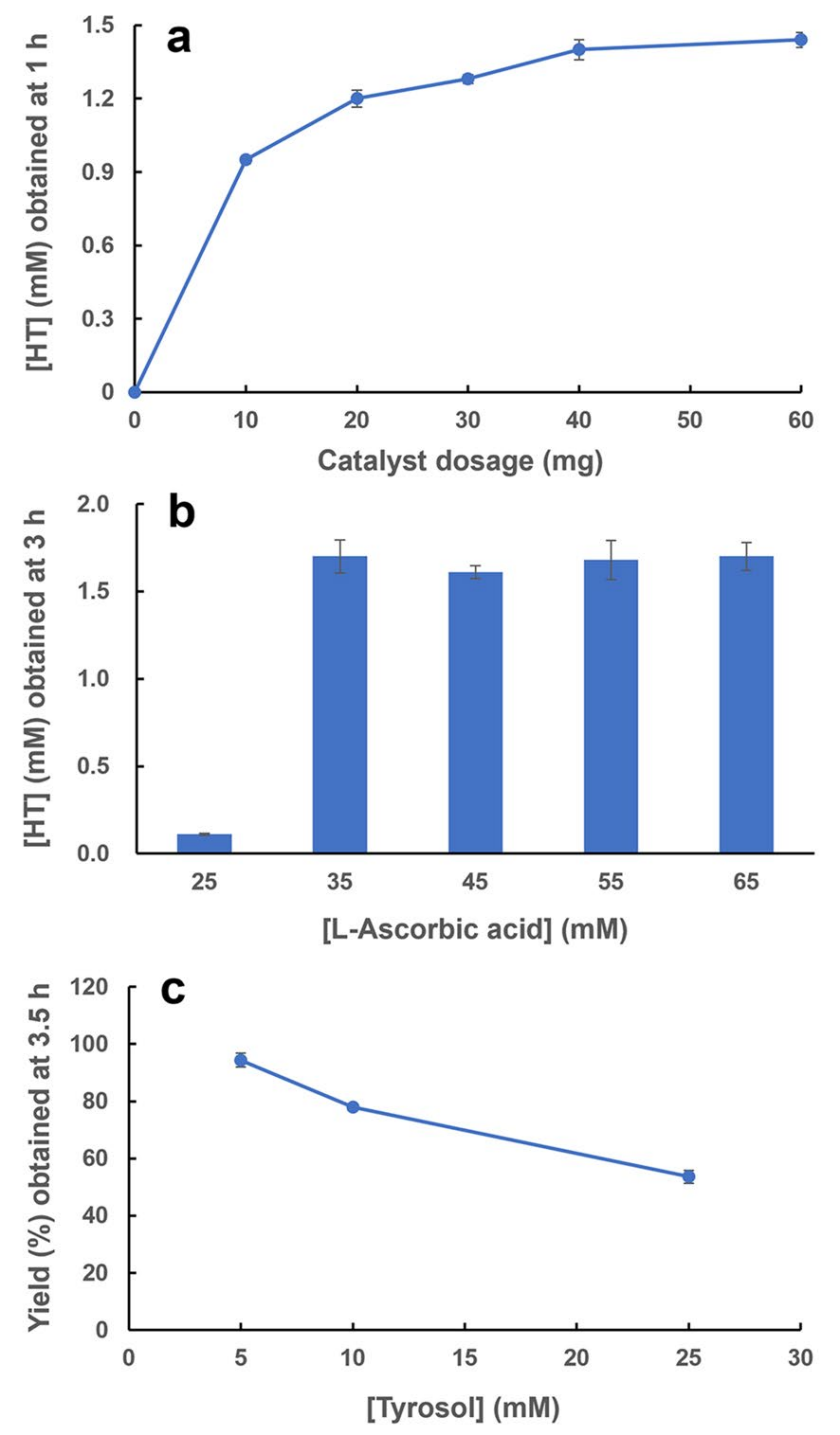
Production of hydroxytyrosol (HT) from tyrosol catalyzed by tyrosinase@HKUST-1 (II): effect of catalyst dosage (**a**), l-ascorbic acid concentration (**b**), and initial tyrosol concentration (**c**) on the formation of HT

The effects of catalyst dosage and concentrations of the reductant and substrate on the product formation are shown in Fig. 6. The initial reaction rate increased as a higher amount of the catalyst was applied (Fig. 6a). Regarding the impact of the reductant l-ascorbic acid (Fig. 6b), its concentration ranging between 35 to 65 mM did not alter the production yield very much; while the rather poor yield obtained in the presence of 25 mM l-ascorbic acid is simply ascribed to the fact that the *o*-diphenol product HT was further oxidized to its *o*-quinone form when the reductant ran out, which was also reflected in the progress curves of the reaction (data not shown). On the other hand, within the same reaction period the yield fell in response to an increase in the substrate concentration (Fig. 6c), which could be partially explained by the competitive inhibition to the enzyme exerted by a high concentration of *o*-diphenols (Deri-Zenaty et al. 2020). A yield as high as 94.4% was achieved in 3.5 h when the original tyrosol concentration was 5.0 mM.

So far there have been only 5 papers in literature regarding the use of tyrosinase as the catalyst, crude or purified, free or immobilized, for synthesizing HT from tyrosol. A comparison is given in Table 1. Espín and coworkers (Espín et al. 2001) were the first to report a successful production of HT from tyrosol with excellent yields and productivities, by using an isolated enzyme, mushroom tyrosinase, as the catalyst (entry 1 in Table 1). The highest productivity obtained by Annunziata et al. (2021) (entry 5 in Table 1) is very much attributed to their smart design of the flow protocol using a gas/liquid biphasic system. Deri-Zenaty et al. (2020) have also reported fairly high productivities (entry 4 in Table 1), as they developed a new way of reducing the *o*-quinone product back to *o*-diphenol by coupling tyrosinase with glucose dehydrogenase, which can continuously generate NADH as the reducing agent in place of l-ascorbic acid. Comparatively speaking, our results are not bad at all, especially when considering that the catalyst we used was prepared by using crude extract from fresh mushrooms, without the need of laborious purification procedures and construction of new metabolic or enzymatic pathways by using synthetic biology tools (Achmon and Fishman 2015; Britton et al. 2019), thus being more practical and cost-effective.

**Table 1:** Production of hydroxytyrosol from tyrosol catalyzed by tyrosinase, crude or purified, free or immobilized, from different sources

	Enzyme source	Catalyst form	[tyrosol]_o_ (mM)	Time (h)	Yield (%)	Productivity (g/L/h)	Refs.
1	*Agaricus bisporus*	Purified, free enzyme	16	4	100	0.62	Espín et al. (2001)
2	*Pycnoporus sanguineus*	Purified, free enzyme	4	1	29	0.18	Halaouli et al. (2005)
3	*Pseudomonas putida* F6	Crude, immobilized in Ca alginate gel	1	2.5	77	0.05	Brooks et al. (2006)
4	*Bacillus megaterium*, expressed in *E. coli*, coupled with glucose dehydrogenase	Purified, immobilized in sol–gel	1	0.5	100	0.31	Deri-Zenaty et al. (2020)
			5	1.5	100	0.51	
			10	2.2	97	0.68	
			50	048	99	0.16	
5	*Agaricus bisporus*	Purified, free enzyme	10	0.5	78	2.40	Annunziata et al. (2021)
6	*Agaricus bisporus*	Crude, immobilized as tyrosinase@HKUST-1	5	3.5	94.4	0.21	This study

**Fig. 7:**
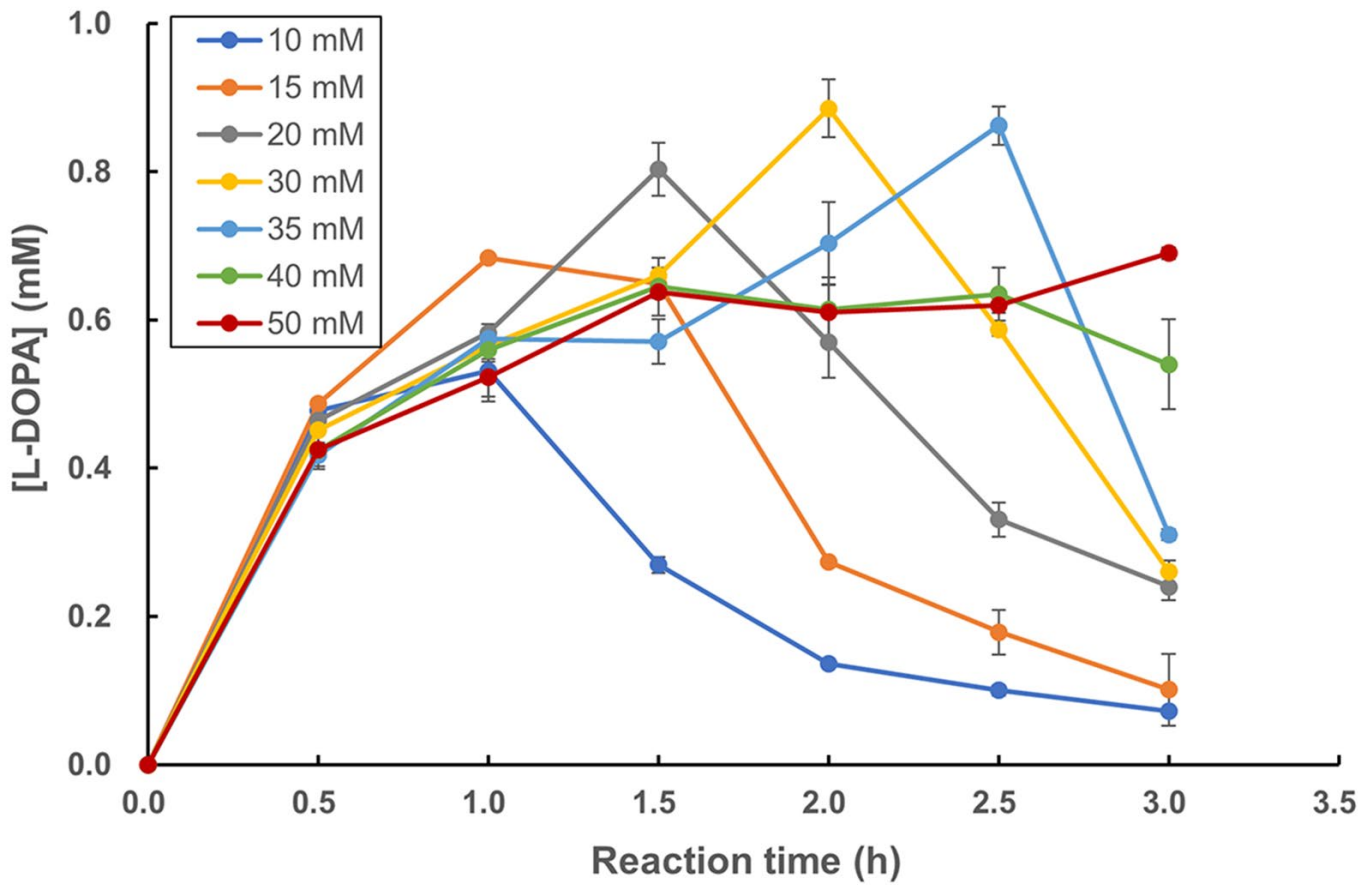
Effect of l-ascorbic acid concentration on production of l-DOPA from l-tyrosine catalyzed by tyrosinase@HKUST-1

Tyrosinase@HKUST-1 is also an efficient catalyst for the synthesis of l-DOPA from its monophenol precursor l-tyrosine. The product was identified by using HPLC (data not shown). Like the above reaction for HT production, increasing the catalyst dosage also led to a higher amount of the product l-DOPA to be formed (data not shown). Figure 7 shows the progress curves of the synthetic reaction in the presence of different concentrations of l-ascorbic acid. It is general that in the presence of the reductant, l-DOPA was more and more produced along with time; but then its level would drop simply because the reduced form of l-ascorbate was increasingly converted to its oxidized form, thus leading to the diminishing of its reducing power. A variation in the reductant concentration (10–50 mM) did not seem to alter the initial reaction rate (see the product formed within 0–1 h). But as l-ascorbate concentration became higher, it took a longer time for the reaction to reach the maximal l-DOPA concentration, accompanied by a higher yield obtained at 3.5 h. This is understandable when taking the reducing power of l-ascorbic acid into consideration. The same phenomenon was also observed in our recent study when TCHCs were applied to catalyze the synthesis of piceatannol from resveratrol (Wei et al. 2020b). In the presence of 30 mM l-ascorbic acid when 50 mg of tyrosinase@HKUST-1 was applied, a yield of 45.5% was achieved within 2.0 h.

To date, many immobilization strategies have been investigated on tyrosinase so as to be used for l-DOPA synthesis. In our previous studies, advanced techniques such as CLEAs (Xu et al. 2012), enzyme-inorganic (Wei et al. 2020b) and enzyme–MOF hybrid composites (Wei et al. 2021) have been utilized to immobilize mushroom tyrosinase for catalyzing the l-DOPA synthesis with excellent yields and productivities. Although the results obtained in this current study were not that satisfactory as compared to our previous ones, they are actually higher than most of others reported in literature (for a comparison see the Table 2 in Wei et al. (2020b)), and better results should be expected after optimization.

## Conclusions

This work offers a third example of immobilizing enzymes on HKUST-1, demonstrating that the simple procedure of mixing the enzyme first with BTC followed by addition of CuAc_2_ is successful for the preparation of tyrosinase@HKUST-1 with super stability. The biocatalyst thus formed is highly effective to catalyze the synthesis of hydroxytyrosol and l-DOPA with great yields and productivities. In a very recent paper, Chen et al. (2021) have proposed a strategy of assembling proteins into biohybrid frameworks with the use of π-conjugated carboxylic acids as the organic linkers, and the authors have claimed that the so-formed biohybrids have highly crystalline framework structure, record-high protein contents, and ultrahigh chemical stability and bioactivity. Our work provides a good support to this. Therefore, as a new immobilization platform HKUST-1 can be introduced to other enzymes for immobilization, and the so-formed tyrosinase@HKUST-1 can be applied to the production of other catecholic products with commercial importance.

## Data Availability

All data generated or analyzed during this study are included in this published article, and are available from the corresponding author on reasonable request.

## Abbreviations

MOFs: Metal–organic frameworks
BTC: Benzene 1,3,5-tricarboxylate
HKUST-1: A type of MOFs with a molecular formula of [Cu_3_(BTC)_2_(H_2_O)_n_]
CLEAs: Cross-linked enzyme aggregates
TCHCs: Tyrosinase–Cu_3_(PO_4_)_2_ hybrid composites
ZIFs: Zeolitic imidazolate frameworks
XRD: X-ray diffraction
FT-IR: Fourier transform infrared spectroscopy
SEM: Scanning electron microscopy
EDS: Energy-dispersive X-ray diffraction spectroscopy
HPLC: High-performance liquid chromatography
TLC: Thin-layer chromatography
l-DOPA: L-3,4-Dihydroxyphenylalanine
HT: Hydroxytyrosol
